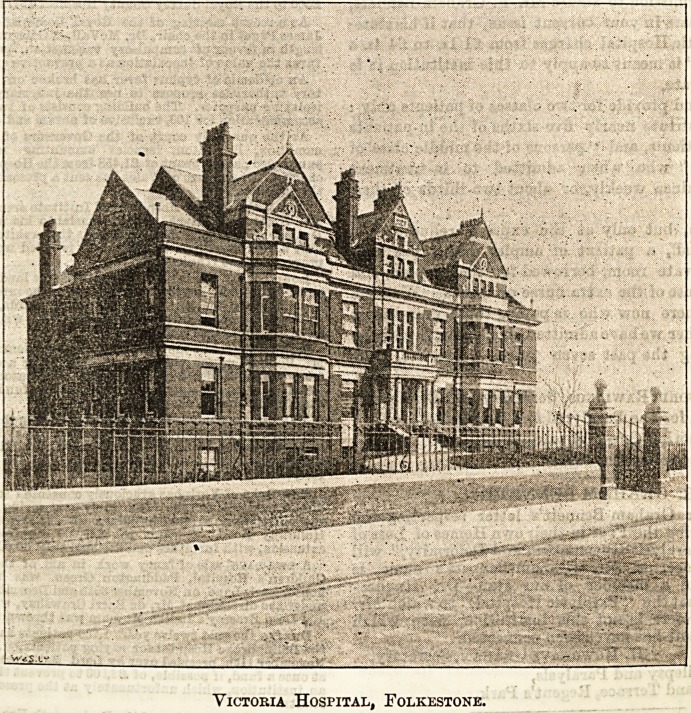# Victoria Hospital, Folkestone

**Published:** 1892-12-10

**Authors:** 


### HOSPITAL CONSTRUCTION.

### VICTORIA HOSPITAL, FOLKESTONE.

A trusted correspondent has recently had the opportunity of viewing this institution, being conducted over it by one of the medical staff, who kindly answered his many inquiries into its working, and who pointed out the chief features of the building.

The hospital was completed about two years ago, the funds being collected in the Jubilee year. It is situated in a very pleasant spot, facing the Radnor Park, and from behind commanding a very fine view of the surrounding hills. From the apparent size of the building, we were surprised to find that it only accommodates twenty-six patients. It was, however, explained to us that the present building is incomplete. It is, in fact, really the central, or administrative, block of a future big hospital. It is proposed, as the needs of the town demand it, to add wings consisting of wards only, and to house the nurses and officers in the rooms at present used as wards. We hope this arrangement may soon obtain, for the present system cannot be regarded as the best.

The four wards now in use are all on the first floor, two for men and two for women, situated on either side of a central passage. The women's wards are at the west end of the passage, and have their own bath rooms and lavatories; the men's wards at the east end have similar arrangements. The wards are pretty and airy, and though we should have preferred "cross" ventilation, the exposed position of the hospital doubtless makes up for this old "infirmary" system. Parquet oak floors and pretty bed coverlets give a very pleasing appearance.

The operation room is one of the best we have seen, and might well serve as a model for similar institutions. The floor is tiled, the light good, and the heating apparatus all that can be desired. The out-patient department is situated in the basement, is small, and low-pitched, but doubtless this also will be remedied when the new wings are built.

A notable feature of the hospital is the fact that wards are set apart for the use of paying patients.

The medical staff consists of a consulting physician, three medical officers, and an assistant surgeon. The medical officers attend to medical, surgical, or obstetric cases indiscriminately. We should have expected, at least, that they would have evoluted into physicians and surgeons. The assistant surgeon has to visit patients at their own homes, for which he receives a salary.

We confess to our surprise at finding that there is no resident house-surgeon. Surely a hospital of this size, where important operations take place, and the emergencies and accidents of a large town are treated, should have a resident house-surgeon. It was pointed out to us that there is telephonic communication to the private houses of the staff, and that the matron makes an excellent house-surgeon. Still, there can be no question but that there are certain classes of cases where the delay of getting a doctor from a distance is fatal to the patient. To mention a few such instances, cases of apparent death from drowning, secondary hæmorrhage, internal hæmorrhage, urgent tracheotomies, "drunk or dying cases."

The nursing staff consists of a matron, head nurse, and four probationers. It is proposed to affiliate a nursing institute with the hospital. The hospital will train the probationers, who, when trained, will be sent out private nursing. We understand that there is no such institution in the town.

Since the opening of the new hospital the number of in-patients has doubled, being now 208 annually. About 2,000 out-patients are also attended to.

VICTORIA HOSPITAL, FOLKESTONE.

That the new building has been opened free of debt speaks well for the business capacity of the committee of management. We understand, however, that the maintenance funds are low, but we cannot think that the Folkestone public, if properly approached, will long allow this to be the case.

The defects which we have pointed out are perfectly remediable, and, though they are such as commonly occur at first in the organization of a country hospital we feel sure that in such an advancing watering-place they will not have a very long lease.

The picture of the hospital given above is from a photograph by Messrs. Lambert, Weston, and Sons.

---

AN addition has been made to Australian periodical literature by the launching on the world of the *Antipodean*, the object of which is to form "a literary link between the mother country and those of her children who are beyond the seas."

## Figures and Tables

**Figure f1:**